# Transient detectable viremia and the risk of viral rebound in patients from the Swiss HIV Cohort Study

**DOI:** 10.1186/s12879-015-1120-8

**Published:** 2015-09-21

**Authors:** Jim Young, Martin Rickenbach, Alexandra Calmy, Enos Bernasconi, Cornelia Staehelin, Patrick Schmid, Matthias Cavassini, Manuel Battegay, Huldrych F. Günthard, Heiner C. Bucher, the Swiss HIV Cohort Study

**Affiliations:** Basel Institute for Clinical Epidemiology and Biostatistics, University Hospital Basel, Basel, Switzerland; Institute of Social and Preventive Medicine, University of Lausanne, Lausanne, Switzerland; Division of Infectious Diseases, University Hospital Geneva, Geneva, Switzerland; Division of Infectious Diseases, Regional Hospital of Lugano, Lugano, Switzerland; Department of Infectious Diseases, Bern University Hospital and University of Bern, Bern, Switzerland; Division of Infectious Diseases and Hospital Epidemiology, Cantonal Hospital St. Gallen, St Gallen, Switzerland; Division of Infectious Diseases, University Hospital Lausanne, Lausanne, Switzerland; Division of Infectious Diseases and Hospital Epidemiology, University Hospital Basel, Basel, Switzerland; Division of Infectious Diseases and Hospital Epidemiology, University Hospital and University of Zürich, Zurich, Switzerland

**Keywords:** HIV, Combination antiretroviral therapy, Viral load, Transient viremia, Adherence

## Abstract

**Background:**

Temporary increases in plasma HIV RNA ('blips') are common in HIV patients on combination antiretroviral therapy (cART). Blips above 500 copies/mL have been associated with subsequent viral rebound. It is not clear if this relationship still holds when measurements are made using newer more sensitive assays.

**Methods:**

We selected antiretroviral-naive patients that then recorded one or more episodes of viral suppression on cART with HIV RNA measurements made using more sensitive assays (lower limit of detection below 50 copies/ml). We estimated the association in these episodes between blip magnitude and the time to viral rebound.

**Results:**

Four thousand ninety-four patients recorded a first episode of viral suppression on cART using more sensitive assays; 1672 patients recorded at least one subsequent suppression episode. Most suppression episodes (87 %) were recorded with TaqMan version 1 or 2 assays. Of the 2035 blips recorded, 84 %, 12 % and 4 % were of low (50–199 copies/mL), medium (200–499 copies/mL) and high (500–999 copies/mL) magnitude respectively. The risk of viral rebound increased as blip magnitude increased with hazard ratios of 1.20 (95 % CI 0.89-1.61), 1.42 (95 % CI 0.96-2.19) and 1.93 (95 % CI 1.24-3.01) for low, medium and high magnitude blips respectively; an increase of hazard ratio 1.09 (95 % CI 1.03 to 1.15) per 100 copies/mL of HIV RNA.

**Conclusions:**

With the more sensitive assays now commonly used for monitoring patients, blips above 200 copies/mL are increasingly likely to lead to viral rebound and should prompt a discussion about adherence.

**Electronic supplementary material:**

The online version of this article (doi:10.1186/s12879-015-1120-8) contains supplementary material, which is available to authorized users.

## Background

Many patients with HIV start combination antiretroviral therapy (cART), achieve a plasma HIV RNA ('viral load') below the level of detection and then experience the occasional 'blip'. A blip is 'a single, low-level but detectable plasma viral load measurement (e.g., 50–1000 copies/mL) that is immediately preceded and followed by a viral load below the limit of detection' [1]. The clinical implications of this transient detectable viremia are unclear. Early studies typically found no association between blips and subsequent viral rebound but were limited by their small sample size (see Table 1 in [2]). However in a recent study of 3550 patients, blips in excess of 500 copies/ml were associated with an increased risk of viral rebound [2].

**Table 1:** Patient characteristics when starting a first suppression episode or a first subsequent suppression episode. Patients had to be antiretroviral treatment naive before achieving viral suppression on a first combination antiretroviral regimen. Viral suppression had to be recorded using a more sensitive assay: ultrasensitive versions of the Amplicor assay (if the lower limit of detection was recorded as <50 copies/ml), the Abbott RealTime assay, and the TaqMan assay versions 1 and 2

Characteristic	Suppression episode	
	First (4094 patients)	First subsequent (1672 patients^a^)
Female (%)	27	34
Injection drug use (%)^b^	9	17
Age (median, years)	40	43
CD4 cell count (median, cells/μL)	430	460
Year (%)		
Before 2005	10	10
2005 to 2009	46	55
After 2009	45	35
Assay (%)^c^		
Roche Amplicor ultrasensitive	12	14
Abbot RealTime	1	1
Roche TaqMan version 1	37	45
Roche TaqMan version 2	50	41
cART class (%)		
NNRTI	42	31
Boosted PI	47	47
Single PI	4	7
Entry or integrase inhibitor	4	7
Other^d^	3	7
Magnitude of first blip (%)		
No blips	78	72
Low (50–199 copies/mL)	19	23
Medium (200–499 copies/mL)	2	4
High (500–999 copies/mL)	1	2
Number of blips (%)		
None	78	72
One	16	20
Two	4	6
Three or more	2	2
Viral rebound (%)	19	33

*cART* combination antiretroviral therapy; *NNRTI* non-nucleoside reverse transcriptase inhibitor; *PI* protease inhibitor

^a^Patients that had a subsequent suppression episode measured using acceptable assays did not always have a first suppression episode measured using acceptable assays

^b^Injection drug use as the most likely mode of HIV infection

^c^First use of each assay in these data: Roche Amplicor ultrasensitive, 1997; Abbot RealTime, 2009; Roche TaqMan version 1, 2002; Roche TaqMan version 2, 2006. Common use of each assay in these data (10^th^ to 90^th^ percentiles): Roche Amplicor ultrasensitive, 2000–05; Abbot RealTime, 2009–12; Roche TaqMan version 1, 2006–09; Roche TaqMan version 2, 2009–13 (current)

^d^Other: More than one PI (other than ritonavir), a PI and an NNRTI, or three nucleoside or nucleotide reverse transcriptase inhibitors. The later was considered cART only if it followed an earlier cART regimen

For more than a decade, the ultrasensitive Roche Amplicor assay has been a standard method of measuring HIV RNA in blood plasma when monitoring patients, with a lower limit of detection of 50 copies/ml [3–6]. The newer assays that have replaced it have lower limits of detection below 50 copies/ml. A number of studies suggest blip magnitudes are higher if measured with either the Roche TaqMan version 1 or version 2 assays relative to the Amplicor assay, although the Abbott RealTime assay may give similar results [6–11]. This necessitates a re-appraisal of the prognostic value of transient detectable viremia.

The causes of transient detectable viremia are also unclear. Plausible explanations include the high variability of assays close to their lower limit of detection [12], residual low level viremia below the usual limit of detection or a release of viral particles from replication in reservoirs [13], vaccination or intercurrent viral infections [14, 15], or less than full adherence to therapy [16]. It seems reasonable to expect that there is no single explanation.

In this study, we consider whether blip magnitude – the level of transient HIV RNA in blood plasma – is predictive of subsequent viral rebound using data from the Swiss HIV Cohort Study (SHCS). We consider the effect of different assays and of adherence on the predictive value of transient detectable viremia in an effort to better understand what a clinician ought to conclude when transient viremia is detected using these more sensitive assays.

## Methods

### Patient selection

The SHCS is a prospective cohort with continuing enrolment of HIV-infected adults [17]. Any HIV-infected patient at least 18 years old can enrol in the SHCS. A signed informed consent is required from all patients. Data collection has been approved by an ethics committee at each participating hospital (Ethikkommission beider Basel, Kantonale Ethikkommission Bern, Comité départemental d'éthique des spécialités médicales et de médecine Genève, Commission cantonale d'éthique de la recherche sur l'être humain Lausanne, Comitato etico cantonale Bellinzona, Ethikkommission des Kantons St. Gallen, Kantonale Ethikkommission Zürich [18]).

For this study, we selected all patients that were antiretroviral treatment naive before achieving viral suppression on their first cART regimen. We defined a first cART regimen as the combination of any three or more antiretroviral drugs (except where all drugs were either nucleoside or nucleotide reverse transcriptase inhibitors). Most patients (95 %) in the SHCS received regimens recommended in clinical guidelines [19]. During 2005 to 2009, the most common first regimens were efavirenz with either tenofovir and emtricitabine or abacavir and lamivudine; lopinavir (boosted with ritonavir) with either tenofovir and emtricitabine or zidovudine and lamivudine; or attazanavir (boosted with ritonavir), tenofovir and emtricitabine [20].

We defined viral suppression as starting at the second of two consecutive viral load measurements <50 copies/ml where the two measurements were at least 30 days apart and made using more sensitive assays. Viral suppression began at the second of these two measurements because our outcome – viral rebound – could not occur between these two measurements [21]. Acceptable assays were modified versions of the ultrasensitive Amplicor assay [4, 22] (if the lower limit of detection was recorded and below 50 copies/ml), the Abbott RealTime assay, and the Roche TaqMan assay versions 1 and 2. These last three assays have lower limits of detection of 40, 40 and 20 copies/ml respectively [6].

### Suppression episodes

A suppression episode consisted of all viral load measurements from the start of suppression until a last measurement to date or until viral rebound, whichever came first. Viral load is measured at cohort visits scheduled every six months but intermediate measurements are also made so that most patients are measured on average once every three months. Patients could contribute more than one suppression episode to our analyses if they again achieved viral suppression after a viral rebound. Some patients contributed a subsequent suppression episode (after a first viral rebound) but did not have a first suppression episode measured using acceptable assays. We defined viral rebound as the first of two consecutive viral load measurements ≥50 copies/mL, where the two measurements were at least 30 days apart, or a single viral load measurement ≥1000 copies/mL [2]. We also considered an alternative definition of viral rebound – the first of two consecutive viral load measurements ≥200 copies/mL [23], where the two measurements were at least 30 days apart, or a single viral load measurement ≥1000 copies/mL.

During each suppression episode, we recorded the magnitude and number of blips. We defined a blip as a viral load 50–999 copies/mL preceded and followed by another measurement <50 copies/mL. Any subsequent viral load measurement of 50–999 copies/mL within 30 days of a blip was considered to be part of the same blip (although we updated the blip magnitude if a subsequent measurement was greater than its predecessor) [2].

### Statistical methods

**Fig. 1:**
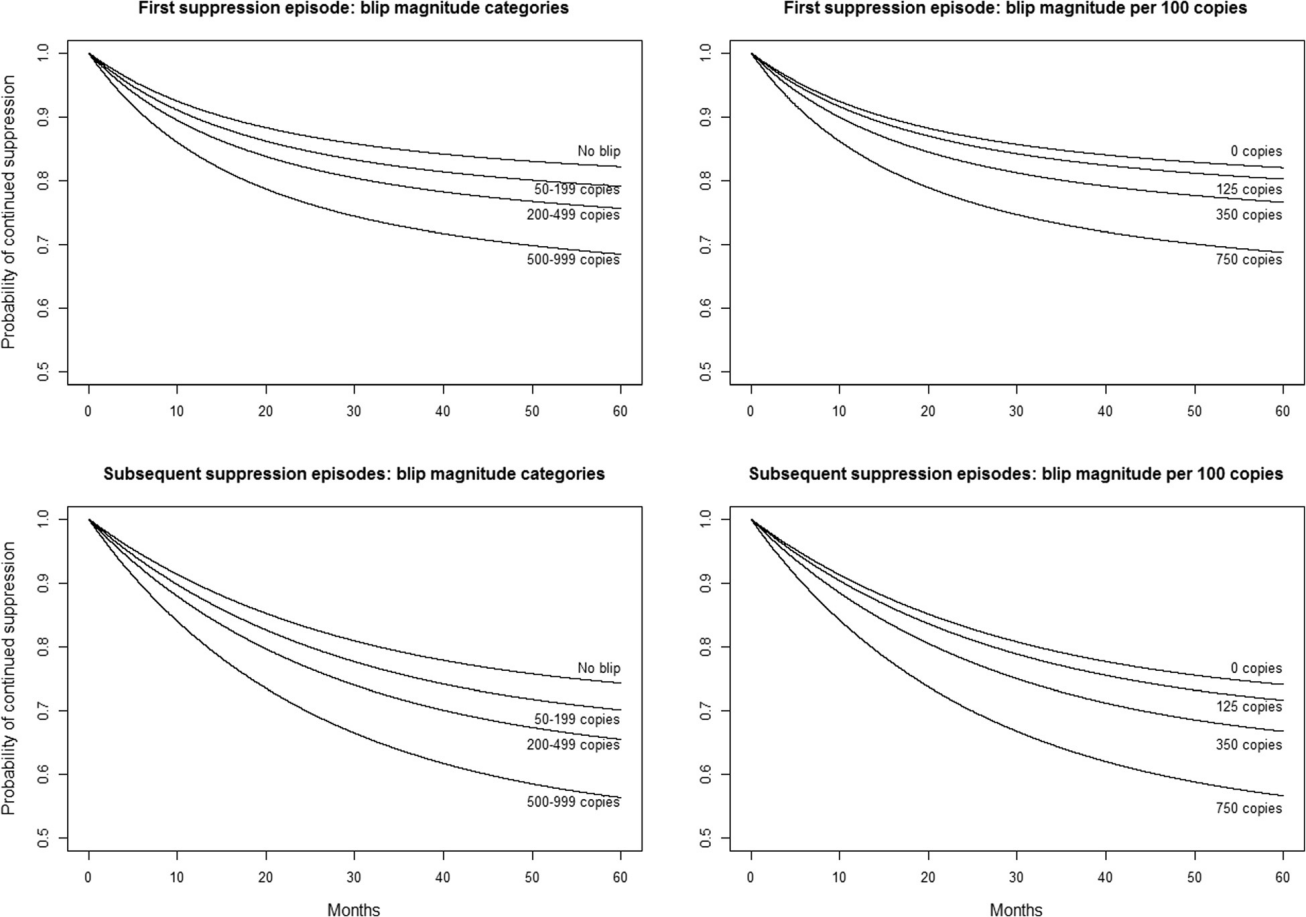
Survival curves with first blip magnitude either in categories (left) or as a continuous measure (right) [24]. Curves are shown for the first suppression episode (top) and for subsequent suppression episodes (bottom). All curves are for the same reference patient: a male who did not acquire HIV though injection drug use, starting a suppression episode in 2005 at the age of 40, on a non-nucleoside reverse transcriptase based regimen, with a CD4 cell count of 350 cells/μL and with HIV RNA measured using an Amplicor ultrasensitive assay

We fitted a variety of proportional hazard models to data from first episodes and used these results to select a suitable model for our analyses of both first and subsequent episodes (Additional file 1: Appendix A). A suitable model should have separate baseline hazard functions for both first and subsequent episodes because although the effect of covariates may be the same in both first and subsequent episodes, the rate of viral rebound is likely to be higher in subsequent episodes (see Fig. 1). The selected model was a generalised linear model for interval censored time to event data [24] with different strata for first and subsequent episodes but we also fitted the gap-time Cox model used in an earlier study [2]. The gap-time model is a standard Cox model stratified by suppression episode and with time reset to zero at the beginning of each new episode (see [25]). However viral rebound is interval censored because it is only known to have occurred at some point between one measurement and the next. The standard Cox model is known to underestimate hazard ratios when measurement error is added to event times [26].

In our first analyses, blips were categorised by magnitude as low (50–199 copies/mL), medium (200–499 copies/mL) or high (500–999 copies/mL) [2]. Indicator variables were used to represent these categories for the first blip per episode; these indicator values were set when a first blip occurred and remained constant until the end of the episode. In subsequent analyses, we represented blip magnitude by a single continuous variable, scaled per 100 copies/mL. In some analyses, this variable was updated to reflect the magnitude of the latest blip in an episode (rather than the first blip) or the cumulative value of blips in an episode to date, or we added another time dependent variable representing the number of blips in the episode to date.

All analyses used the same set of covariates: gender, injection drug use as the most likely mode of infection, age at the beginning of the suppression episode, the year the suppression episode began, the assay used to measure the blip, cART categories and CD4 cell count. These last two covariates were updated whenever their values changed within a suppression episode. We did not censor patients if they stopped taking cART because such censoring could be informative; rather we included a category for 'no cART'. These covariates were all used in an earlier study [2] except current CD4 cell count which we added to our model because this is a strong predictor of HIV progression even in patients with a suppressed viral load [27]. CD4 cell count was represented by a linear spline with a knot at 200 cells/μL and scaled per 100 cells/μL [27]. To estimate the effect of covariates on the predictive value of a blip, we added appropriate interaction terms to our analyses (rather than carry out separate analyses for different values of a covariate) [28].

All models were fit in SAS 9.3. Model parameters are reported as the estimated hazard ratio (HR) and its 95 % confidence interval (95 % CI).

## Results

### Study population

As at May 2014, 4094 antiretroviral naive patients in the SHCS started treatment with a cART regimen and recorded a first episode of viral suppression using more sensitive assays; 1672 of these patients later recorded a subsequent episode of viral suppression on cART using more sensitive assays. The median length of first and subsequent suppression episodes was 2.9 [interquartile range, IQR, 1.3 to 5.0] and 2.3 [IQR 1.0 to 4.7] years, respectively. The median time between RNA measurements in first and subsequent suppression episodes was 3.3 [IQR, 2.8 to 4.4] and 3.3 months [IQR 2.9 to 4.4], respectively. Most suppression episodes (87 %) were recorded with TaqMan version 1 or 2 assays (Table 1). Patients typically started a first suppression episode with cART based on either a boosted protease inhibitor (PI, 47 %) or a non-nucleoside reverse transcriptase inhibitor (NNRTI, 42 %), but the latter was less common in subsequent episodes (30 %). The rate of blips was 8.7 per 100 person years in first suppression episodes and 12.3 per 100 person years in subsequent suppression episodes. The rate of viral rebound was 5.6 per 100 person years in first suppression episodes and 10.6 per 100 person years in subsequent suppression episodes. In first suppression episodes, 19 % of 785 rebounds were preceded by a blip; in subsequent suppression episodes, 22 % of 695 rebounds were preceded by a blip.

Of the 2035 blips recorded, 84 %, 12 % and 4 % were of low, medium and high magnitude respectively. The time between a blip and the next viral load measurement decreased with increasing blip magnitude: a median of 2.8, 1.9 and 1.5 months for low, medium and high magnitude blips respectively. A change in cART between a blip and the next viral load measurement was more likely as blip magnitude increased: 2.4 %, 4.6 % and 6.1 % of low, medium and high magnitude blips, respectively, were followed by a change in cART class. Of the 57 changes in cART class, common changes were from boosted PI based therapy to either NNRTI (25 %) or entry or integrase inhibitor (14 %) based therapy, or from NNRTI based therapy to either boosted PI (9 %) or entry or integrase inhibitor (11 %) based therapy.

### Blip magnitude and subsequent viral rebound

When fit to first and subsequent episodes, both interval censoring and gap-time Cox models suggest a gradual increase in the risk of viral rebound with increasing blip magnitude rather than a threshold effect (Table 2). Under our model, estimates are: HR 1.20 (95 % CI 0.89 to 1.61), HR 1.42 (95 % CI 0.96 to 2.19), HR 1.93 (95 % CI 1.24 to 3.01) for low, medium and high magnitude blips respectively. Fitting these models to data from first episodes only led to similar but less precise estimates (Additional file 1: Appendix A). Replacing the three categories of blip magnitude with a continuous variable makes it easier to test whether the association between blip magnitude and viral rebound varies with other factors. For example, the relative risk of viral rebound with increasing blip magnitude, estimated in our model to be HR 1.09 (95 % CI 1.03 to 1.15) per 100 copies/mL of HIV RNA, was similar in both first and subsequent suppression episodes (HR 1.11, 95 % CI 1.03 to 1.19, and HR 1.07, 95 % CI 1.00 to 1.15, per 100 copies/mL respectively). In the gap-time model, the relative risk of viral rebound with increasing blip magnitude was estimated to be HR 1.08 (95 % CI 1.03 to 1.14) per 100 copies/mL. Survival curves show that this model simplification, from categories to a continuous blip magnitude, did not materially alter the predicted probabilities of viral rebound for a reference patient (Fig. 1). Note that while blip magnitude appears to have a similar association with viral rebound in both first and subsequent suppression episodes, the baseline probability of viral rebound is greater in subsequent suppression episodes than in first suppression episodes (Fig. 1). Using an alternative definition of viral rebound – the first of two consecutive viral load measurements ≥200 copies/mL or a single viral load measurement ≥1000 copies/mL – reduced the number of rebounds by 24 % to 600 in first suppression episodes and by 40 % to 422 in subsequent suppression episodes. This attenuated HR estimates for blip magnitude categories and reduced their precision (Additional file 2: Appendix B) but the estimate for a continuous variable (HR 1.07, 95 % CI 1.01 to 1.13, per 100 copies/mL of HIV RNA) suggests that even with this alternative definition, there is still a gradual increase in the risk of viral rebound with increasing blip magnitude.

**Table 2:** Estimates of associations between covariates and subsequent viral rebound in models fit to data from both first and subsequent suppression episodes

Covariate	Hazard ratio (95 % confidence interval)	
	Model for interval censored data [24]	Gap-time Cox model [2]
Magnitude of first blip (reference no blips)		
Low (50–199 copies/mL)	1.20 (0.89, 1.61)	1.03 (0.77, 1.38)
Medium (200–499 copies/mL)	1.42 (0.96, 2.19)	1.25 (0.86, 1.82)
High (500–999 copies/mL)	1.93 (1.24, 3.01)	1.69 (1.10, 2.60)
Female	1.16 (1.03, 1.30)	1.12 (1.00, 1.25)
Injection drug use^a^	1.21 (1.04, 1.41)	1.25 (1.08, 1.45)
Age (per 10 years)	0.91 (0.86, 0.97)	0.89 (0.84, 0.95)
Calendar year episode began (per year)	0.92 (0.90, 0.94)	0.93 (0.91, 0.95)
Assay (reference Roche Amplicor ultrasensitive)		
Abbot RealTime	2.19 (0.65, 7.41)	2.46 (0.81, 7.47)
Roche TaqMan version 1	0.90 (0.65, 1.25)	0.87 (0.63, 1.19)
Roche TaqMan version 2	1.31 (0.93, 1.86)	1.23 (0.88, 1.72)
cART regimen (reference NNRTI based)		
Boosted PI	1.85 (1.62, 2.10)	1.76 (1.55, 2.01)
Single PI^b^	1.73 (1.39, 2.16)	1.68 (1.35, 2.09
Entry or integrase inhibitor^c^	1.98 (1.63, 2.41)	1.77 (1.45, 2.15)
None^d^	10.6 (8.17, 13.7)	8.70 (6.87, 11.0)
CD4 cell count (per 100 cells/μL)^e^		
0 to <200	0.74 (0.57, 0.97)	na
≥ 200	1.01 (0.98, 1.03)	na
RNA tests per year (reference >6)^e^		
≤ 3	na	0.31 (0.23, 0.42)
3- ≤ 4	na	0.36 (0.27, 0.48)
4- ≤ 6	na	0.47 (0.27, 0.48)

*cART* combination antiretroviral therapy; *NNRTI* non-nucleoside reverse transcriptase inhibitor; *PI* protease inhibitor; *na* not applicable

^a^Injection drug use as the most likely mode of HIV infection

^b^Also includes regimens with three nucleoside or nucleotide reverse transcriptase inhibitors - such regimens were considered cART if they followed another earlier cART regimen

^c^Also includes regimens with more than one PI (other than ritonavir), or with a PI and an NNRTI - all these regimens were mostly used as salvage regimens during this era

^d^The cART regimen was updated whenever its value changed within a suppression episodes. A patient not on cART was highly likely to experience viral rebound

^e^The gap-time Cox model in [2] has the number of RNA tests per year as a covariate but not CD4 cell count. The number of RNA tests per year is not an appropriate covariate in models for interval censored data – see, Additional file 1: Appendix A. Current (time updated) CD4 cell count was added to the model for interval censored data because it is a strong predictor of HIV progression even in patients with a suppressed viral load [27]

### Blip magnitude and assays

There was some evidence that viral rebound was associated with blips measured using the TaqMan version 2 assay relative to the Amplicor ultrasensitive assay (HR 1.31, 95 % CI 0.93 to 1.86, Table 2). However blip magnitude was not appreciably more predictive of viral rebound with this assay than with other assays (HR 1.11, 95 % CI 0.97 to 1.26, per 100 copies/mL using the TaqMan version 2 assay, and HR 1.08, 95 % CI 1.02 to 1.15, per 100 copies/mL using other assays). And low magnitude blips were not appreciably less predictive of viral rebound with this assay than with other assays, although this comparison lacks power (HR 1.11, 95 % CI 0.60 to 2.04, using the TaqMan version 2 assay, and HR 1.21, 95 % CI 0.90 to 1.63, using other assays).

### Blip magnitude and adherence

**Fig. 2:**
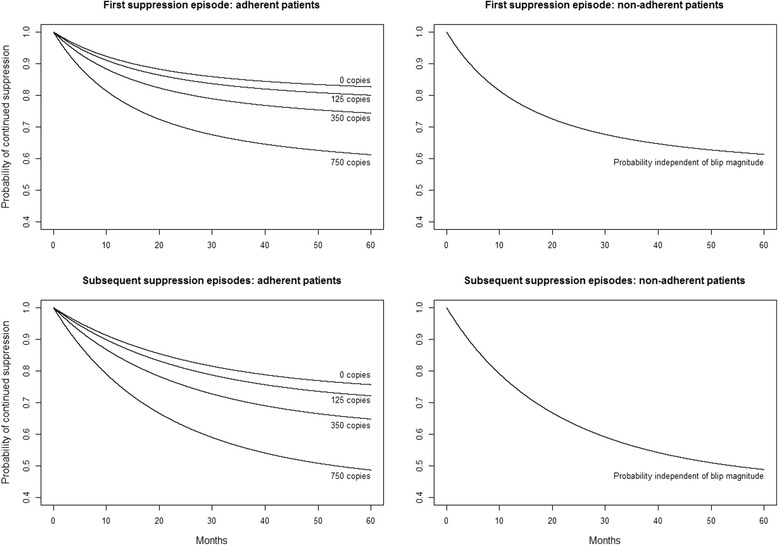
Survival curves given a patient reporting either complete adherence to antiretroviral therapy (left) or non-adherence to antiretroviral therapy (right) [24]. Curves are shown for the first suppression episode (top) and for subsequent suppression episodes (bottom). All curves are for the same reference patient: a male who did not acquire HIV though injection drug use, starting a suppression episode in 2005 at the age of 40, on a non-nucleoside reverse transcriptase based regimen, with a CD4 cell count of 350 cells/μL and with HIV RNA measured using an Amplicor ultrasensitive assay

Routine collection of adherence data started in the SHCS in May 2003. We defined non-adherent patients as those who reported missing either two or more doses of an antiviral medication in a month [29]. In an analysis of data collected after May 2003, the risk of viral rebound with increasing blip magnitude was similar both in those reporting adherence and in those reporting non-adherence at the time of the blip (HR 1.04, 95 % CI 0.94 to 1.15, and HR 1.09, 95 % CI 0.93 to 1.27, per 100 copies/mL respectively). However, in an analysis with time updated non-adherence as a covariate, both reporting non-adherence and not responding to questions on adherence were associated with a higher risk of viral rebound (HR 2.58, 95 % CI 2.07 to 3.22, and HR 1.51, 95 % CI 1.34 to 1.69, respectively). In adherent patients, blip magnitude was predictive of viral rebound (HR 1.13, 95 % CI 1.05 to 1.23, per 100 copies/mL); in non-adherent patients, it was not (HR 0.96, 95 % CI 0.80 to 1.17, per 100 copies/mL). The strong association between non-adherence and viral rebound (Fig. 2) is consistent with the strong association between stopping cART and viral rebound (HR 10.6, 95 % CI 8.17 to 13.7, Table 2).

### More than one blip per suppression episode

In first suppression episodes, 6 % of patients had more than one blip; in subsequent suppression episodes, 8 % of patients had more than one blip (Table 1). The magnitude of the first blip in a suppression episode was as predictive of viral rebound (HR 1.09, 95 % CI 1.03 to 1.15, per 100 copies/mL) as the magnitude of the most recent blip or the cumulative magnitude of blips during an episode (HR 1.10, 95 % CI 1.04 to 1.16, and HR 1.10, 95 % CI 1.06 to 1.14, per 100 copies/mL respectively). However the risk of viral rebound increased with both the magnitude of the first blip and the number of blips per suppression episode (HR 1.28, 95 % CI 1.12 to 1.45, per blip).

## Discussion

Earlier conclusions based on RNA measurements made using Amplicor ultrasensitive and bDNA assays [2] seem to broadly apply to our data where most RNA measurements were made with TaqMan version 1 or 2 assays. We use definitions and methods consistent with this earlier study so results can be directly compared. However our data show a gradual increase in the relative risk of viral rebound with increasing blip magnitude (HR 1.09, 95 % CI 1.03 to 1.15, per 100 copies/mL of HIV RNA), rather than a threshold effect. The threshold of 500 copies/mL suggested by this earlier study [2] corresponds to an estimated hazard ratio of nearly 2 in our data but a lower threshold of 200 copies/mL still leads to an increase in relative risk with an estimated hazard ratio of nearly 1.5.

Agreement between more sensitive assays is poor below 200 copies/mL [6, 12]. A number of studies have shown that blip magnitudes are higher if measured with either the TaqMan version 1 or version 2 assays relative to the Amplicor assay [7–10]. This has led to the suggestion that with more sensitive assays, 100 to 200 copies/mL might be a more appropriate threshold for concern than 50 copies/mL [8, 9] and guidelines now define virologic failure as a persistent plasma RNA of 200 copies/mL or more [23].

Our analyses lack the power to precisely estimate individual associations between blip magnitude and viral rebound for each assay. It is reasonable to expect that low magnitude blips will be less predictive of viral rebound when made with a TaqMan version 2 assay given reports that more low magnitude blips are detected with this assay [6, 9–11]. Our estimates are consistent with this expectation but are too imprecise to be definitive. The increased sensitivity of the TaqMan version 2 assay, however, does not seem material in these data, in contrast to studies suggesting such effects might be important [9, 10], because blip magnitudes measured with this assay were not dramatically more or less predictive of viral rebound than blip magnitudes measured with other assays.

It is important to distinguish between transient viremia and persistent low level viremia [30]. Recent studies in SHCS and other data show that persistent low level viremia, typically defined as two or more consecutive detectable viral load measurements below 1000 copies/mL, is associated with an increased risk of virologic failure [31–33]. Even persistent viremia below 50 copies/mL may increase the risk of viral rebound [34]. In our data, the time between a blip and the next viral load measurement decreased with increasing blip magnitude and this suggests clinicians were responding appropriately to low but detectable measurements to determine whether such measurements were the first sign of virologic failure. Persistent low level viremia becomes an event in these analyses through our definition of viral rebound. While it is possible that blips are a precursor to persistent low-level viremia [1], blips typically do not lead to drug resistance [8, 9, 16, 35].

Our results are consistent with the hypothesis that the association between blip magnitude and viral rebound largely arises through periodic non-adherence [16, 36, 37]. First, where a patient was known to be non-adherent, knowledge of blip magnitude was then redundant (Fig. 2). Second, the magnitude of the first blip in a suppression episode was no less predictive of subsequent viral rebound than either the magnitude of the most recent blip in an episode or the cumulative magnitude of blips in an episode; yet the number of blips in the episode was additionally predictive of viral rebound. The lack of evidence either for a stronger effect with updated measurements [38] or for a stronger cumulative effect [39] is consistent with blips being a marker for periodic non-adherence rather than due to random variation in residual viral replication below the usual limit of detection [13, 40]. That is, in these data the amount of replication during a suppression episode seems less important than whether there was replication or not. The increasing risk of rebound with increasing blip magnitude might represent a probabilistic separation between residual replication below 10 copies/mL [40, 41] found in many suppressed patients and sporadically detected and over-estimated by more sensitive assays, in particular, the TaqMan version 2 [11, 12], and replication resulting from non-adherence, with the latter much more likely to be associated with viral rebound than the former.

Viral rebound was also more likely in females, in younger patients and in those infected through injection drug use – as in an earlier study [2]. Increases in CD4 cell count were associated with a lower risk of viral rebound but only while patients had a CD4 cell count below 200 cells/μL, consistent with other studies where a CD4 cell count of 200 cells/μL proved to be an important threshold [27]. Viral rebound was more likely with the use of either boosted PI based cART or entry or integrase inhibitor based cART. When these data were collected, such regimens were typically prescribed to patients thought more prone to non-adherence or to patients who had experienced virologic failure on other regimens. However viral rebound was both more likely with the use of single PI cART and less likely with increasing calendar time and this suggests that newer regimens have reduced the risk of viral rebound.

The strengths of this study are data collected with more sensitive assays and data on reported adherence to cART, the use of statistical models appropriate for interval censored time to event data and the combining of data from first and subsequent suppression episodes (given that it seems appropriate to do so). Limitations include a lack of power to estimate assay specific associations — in particularly, there was little use of Abbott RealTime assay — and we expect there will be some differences between more sensitive assays in the predictive value of increasing blip magnitude [42]. Self-reported adherence overestimates adherence but there is a strong association between self-reported adherence and virological outcomes [29, 43]. Misclassification of non-adherent patients as adherent could lead to an underestimate of the association between non-adherence and viral rebound but is unlikely to result in residual confounding in the association between blip magnitude and viral rebound because adjustment for time dependent non-adherence did not change the point estimate for the effect of blip magnitude (from HR 1.09, 95 % CI 1.03 to 1.15, to HR 1.09, 95 % CI 1.01 to 1.18 per 100 copies/mL of HIV RNA) [44, 45]. We did not consider the frequency of blips, and we expect that blips are more likely both with more sensitive assays and with more intensive RNA monitoring in patients with a higher risk of disease progression [2]. In this study we focus on what clinicians ought to conclude when they detect a blip with the more sensitive assays now commonly used for monitoring patients.

## Conclusion

Taken together, this and other recent studies suggest that with more sensitive assays, blips in excess of 200 copies/mL are increasingly likely to be due to non-adherence, rather than due to either random variation in residual viremia or assay measurement error, and are therefore a reason for clinicians to discuss adherence with their patients.

### Availability of data and materials

Not applicable.

## Additional files

Additional file 1: Appendix A. Alternative proportional hazards models. (PDF 286 kb) Additional file 2: Appendix B. An alternative definition of viral rebound. (PDF 273 kb)

## Abbreviations

cART: Combination antiretroviral therapy
SHCS: Swiss HIV Cohort Study
HR: Hazard ratio
CI: Confidence interval
IQR: Interquartile range
PI: Protease inhibitor
NNRTI: Non-nucleoside reverse transcriptase inhibitor
