# Supplementary material for: Transient detectable viremia and the risk of viral rebound in patients from the Swiss HIV Cohort Study
Source: BMC Infect Dis. 2015 Sep 21;15:382. doi: 10.1186/s12879-015-1120-8 (PMC4578247; doi:10.1186/s12879-015-1120-8)
Supplement: Additional file 1: Appendix A. — Alternative proportional hazards models. (PDF 286 kb) [file 12879_2015_1120_MOESM1_ESM.pdf]

Appendix A: Alternative proportional hazards models

Methods

We considered a variety of proportional hazards models with which to estimate the association between blip magnitude and the time to viral rebound:

1. The continuous time Cox model used in an earlier study, with the yearly rate of viral load measurements as a covariate [1].
2. A discrete time Cox model, where the baseline hazard is represented by a set of intercept parameters, one for each visit where a viral load measurement was made [2].
3. A continuous time Cox model, where the baseline hazard is represented by a second degree polynomial fit to the mid-point of the interval between successive measurements [3].
4. A Weibull proportional hazards model for interval censored events and time dependent covariates [4].

Model 1 assumes the time of viral rebound is known exactly; however viral rebound is interval censored and is only known to have occurred at some point between one measurement and the next. Adding the yearly rate of viral load measurements as a covariate does not necessarily solve this problem. The value of this covariate tends to decrease the longer patients remain suppressed, as monitoring becomes less intensive in successfully treated patients. So this covariate is, at least in part, a proxy measure for the length of time the patient remains in the analysis, and then by definition viral rebound will be less likely for lower values of this covariate (as seen in Table 2 and in an earlier study [1]). Proportional hazards models are easier to interpret when the two components of the model are separate so that the baseline hazard depends on time but not on covariate values, and the effect of covariates depends on their values but not on time.

Model 2 is exact for interval censored events if the interval between subsequent measurements is the same for all patients. Where it is not, Model 2 approximates the baseline hazard by adding an offset to the model equal to the log of the time between each measurement (so

that rebound at a subsequent visit is more likely as the interval between visits increases) and robust standard errors are needed [2]. However Model 2 may be more appropriate if all measurements were made at regular scheduled cohort visits but less appropriate here given there were also intermediate measurements made between cohort visits for most but not all patients.

Model 3 is the same as Model 2 except the baseline hazard is approximated by a smooth function with only two parameters (Model 3A) and this hazard is estimated at interval mid-points rather than at each visit. A second degree polynomial should provide an adequate baseline hazard function if the baseline hazard is a regular function of time and this was checked by fitting a more flexible function (Model 3B).

Models 1 to 3 can be fit to time to event data in the counting process format and can have different baseline hazard functions for first and subsequent suppression episodes. Models 2 to 4 are appropriate for interval censored event data. Model 4 assumes the baseline hazard function follows a Weibull distribution; this may be adequate if the baseline hazard is a regular function of time. However this model only allows for a single baseline hazard function and so we cannot fit this model to data from both first and subsequent suppression episodes.

A suitable model for these data should have separate baseline hazard functions for both first and subsequent episodes because although the effect of covariates may be the same in both first and subsequent episodes, the rate of viral rebound is likely to be higher in subsequent episodes. We therefore compare results from these models when fit to data from first episodes only and used these results to select a suitable model for the analysis of data from both first and subsequent episodes.

Results

The median length of first suppression episodes was 2.9 [IQR 1.3 to 5.0] years. As expected, there was an association between the number of RNA tests per year and the length of the first suppression episode: for patients with ≤3, 3-≤4, 4-≤6 and > 6 RNA measurements per year, the median episode

length was 4.0 [IQR 2.5 to 5.8], 3.4 [IQR 1.8 to 5.4], 2.2 [IQR 1.0 to 4.0] and 0.4 [IQR 0.2 to 0.7] years, respectively.

The interval between successive RNA measurements was 3.3 [IQR 2.8 to 4.4] months during first suppression episodes. This interval increased the longer the patient remained suppressed, particularly with measurements made by private physicians (Table A1). This was due to less frequent intermediate RNA measurements because the interval between RNA measurements made at scheduled cohort visits did not vary with either the setting or the length of time suppressed.

Model 3 seemed an adequate model for these data. Using data from just first episodes, Models 3 and 4 gave similar estimates of associations between blip magnitude and subsequent viral rebound (Table A2). With Model 3, estimates were also similar when the baseline hazard function was represented by a more flexible cubic spline with five knots (Model 3B) rather than by a second order polynomial (Model 3A). Model 3 has a non-parametric baseline hazard function but approximates how this function behaves in the intervals between visits. Model 4 has a parametric baseline hazard function; this could be less flexible than non-parametric alternatives but it provides an exact likelihood for interval censored events.

The similar estimates from Models 3A and 3B suggest that the baseline hazard function is not a complicated function of time. In this case, estimates from Model 4 should be reliable. Recent simulations support this conclusion and recommend the use of parametric proportional hazard models for interval censored data [5]. Relative to Model 4, Models 1 and 2 appear to under and over estimate, respectively, although all estimates lead to the same clinical conclusions and each model shows an increase in the relative risk of viral rebound with increasing blip magnitude. Model 1 is known to underestimate hazard ratios when measurement error is added to event times [6].

We therefore used Model 3A to estimate the association between blip magnitude and the time to viral rebound in data from both first and subsequent suppression episodes.

Table A1. Median [interquartile range, IQR] interval between successive RNA measurements in first suppression episodes cross-classified by setting and time since starting combination antiretroviral therapy (cART).

Setting	Time since starting cART (years)	Interval between successive RNA measurements (months)			
		All measurements		Cohort visit measurements	
		Median	IQR	Median	IQR
Main hospitals	0 to <2	3.0	[2.4 to 3.5]	6.2	[5.8 to 6.8]
	2 to <5	3.2	[2.8 to 4.0]	6.3	[5.8 to 7.0]
	5 to <10	3.2	[2.9 to 4.2]	6.3	[5.8 to 7.0]
	10 to <20	3.2	[2.9 to 4.2]	6.3	[5.8 to 7.2]
Regional hospitals	0 to <2	3.1	[2.9 to 3.7]	6.3	[5.8 to 6.8]
	2 to <5	3.4	[3.0 to 5.4]	6.3	[6.0 to 7.0]
	5 to <10	3.9	[3.0 to 6.0]	6.3	[6.0 to 7.2]
	10 to <20	3.7	[3.0 to 6.0]	6.4	[6.0 to 7.1]
Private physicians	0 to <2	3.4	[2.8 to 4.4]	6.3	[5.8 to 7.3]
	2 to <5	4.1	[3.1 to 6.0]	6.4	[5.8 to 7.5]
	5 to <10	4.4	[3.3 to 6.1]	6.3	[5.9 to 7.5]
	10 to <20	4.7	[3.4 to 6.1]	6.3	[5.8 to 7.4]

Table A2. Estimates of associations between the magnitude of the first blip in a suppression episode and subsequent viral rebound when models were fit to data from first suppression episodes only.

Model[a]	First blip magnitude: hazard ratio (95% confidence interval) relative to no blip		
	50–199 copies/mL	200–499 copies/mL	500–999 copies/mL
1	1.01 (0.70, 1.45)	1.14 (0.68, 1.91)	1.94 (1.02, 3.69)
2	1.36 (0.90, 2.07)	1.55 (0.83, 2.90)	2.24 (1.12, 4.46)
3A	1.21 (0.81, 1.80)	1.31 (0.69, 2.47)	2.17 (1.14, 4.15)
3B	1.22 (0.82, 1.83)	1.30 (0.68, 2.47)	2.14 (1.12, 4.10)
4	1.12 (0.77, 1.61)	1.29 (0.77, 2.17)	2.03 (1.07, 3.85)

[a] All models used the same covariates as in Table 2: gender, transmission by injection drug use, age at the start of the suppression episode, the year the suppression episode began, the assay used to measure the blip, and time updated cART categories. Model 1 also included covariates for the yearly rate of viral load measurements (as in [1]); in all other models, these three covariates were dropped and two covariates were added to represent time updated CD4 cell count.

References

1. Grennan JT, Loutfy MR, Su D, Harrigan PR, Cooper C, Klein M, et al. Magnitude of virologic blips is associated with a higher risk for virologic rebound in HIV-infected individuals: a recurrent events analysis. J Infect Dis 2012; 205:1230-8.

2. Carlin JB, Wolfe R, Coffey C, Patton GC. Analysis of binary outcomes in longitudinal studies using weighted estimating equations and discrete-time survival methods: prevalence and incidence of smoking in an adolescent cohort. Stat Med 1999; 18:2655-79.

3. Smith PJ, Thompson TJ, Jereb JA. A model for interval-censored tuberculosis outbreak data. Stat Med 1997; 16:485-96.

4. Sparling YH, Younes N, Lachin JM, Bautista OM. Parametric survival models for interval-censored data with time-dependent covariates. Biostatistics 2006; 7:599-614.

5. Gong Q, Fang L. Comparison of different parametric proportional hazards models for interval-censored data: a simulation study. Contemp Clin Trials 2013; 36:276-83.

6. Meier AS, Richardson BA, Hughes JP. Discrete proportional hazards models for mismeasured outcomes. Biometrics 2003; 59:947-54.
