# Supplementary material for: Transient detectable viremia and the risk of viral rebound in patients from the Swiss HIV Cohort Study
Source: BMC Infect Dis. 2015 Sep 21;15:382. doi: 10.1186/s12879-015-1120-8 (PMC4578247; doi:10.1186/s12879-015-1120-8)
Supplement: Additional file 2: Appendix B. — An alternative definition of viral rebound. (PDF 273 kb) [file 12879_2015_1120_MOESM2_ESM.pdf]

Appendix B: An alternative definition of viral rebound

Methods

In the main analysis, we defined viral rebound as the first of two consecutive viral load measurements ≥50 copies/mL, where the two measurements were at least 30 days apart, or a single viral load measurement ≥1000 copies/mL. We did this so our definitions were consistent with an earlier study [1]. However guidelines define viral rebound as a confirmed HIV RNA ≥200 copies/mL [2]. Hence in a set of sensitivity analyses, we re-defined viral rebound as the first of two consecutive viral load measurements ≥200 copies/mL, where the two measurements were at least 30 days apart, or a single viral load measurement ≥1000 copies/mL.

Results

Using this alternative definition of viral rebound (original results in parentheses), the rate of viral rebound was 4.1 (5.6) per 100 person years in first suppression episodes and 7.7 (10.6) per 100 person years in subsequent suppression episodes. In first suppression episodes, 28% (19%) of 600 (785) rebounds were preceded by a blip; in subsequent suppression episodes, 28% (22%) of 422 (695) rebounds were preceded by a blip.

This change in definition decreased the number of rebounds by 24% in first suppression episodes and by 39% in subsequent suppression episodes. The change attenuated estimates for blip magnitude categories and reduced their precision, particularly when using data from first suppression episodes only (Table B1). However, when using data from both first and subsequent episodes, a gradual increase in the relative risk of viral rebound with increasing blip magnitude is still apparent and the estimate of this increase (HR 1.07, 95% CI 1.01 to 1.13, per 100 copies/mL of HIV RNA) is similar to the estimate in the main analysis (HR 1.09, 95% CI 1.03 to 1.15, and per 100 copies/mL of HIV RNA).

Table B1. Estimates of associations between the magnitude of the first blip in a suppression episode and subsequent viral rebound when using an alternative definition of viral rebound.

Data and exposure variable	Hazard ratio (95% confidence interval) [a]	
	Model for interval censored data [3]	Gap-time Cox model [1]
Data from first episodes only		
Magnitude of first blip per 100 copies/mL	1.03 (0.94, 1.14)	1.04 (0.96, 1.12)
Magnitude of first blip (reference no blips)		
Low (50 - 199 copies/mL)	1.16 (0.71, 1.91)	1.00 (0.70, 1.44)
Medium (200 - 499 copies/mL)	1.19 (0.40, 3.56)	1.15 (0.73, 1.83)
High (500 - 999 copies/mL)	1.16 (0.60, 2.23)	1.07 (0.53, 2.18)
Data from first and subsequent episodes		
Magnitude of first blip per 100 copies/mL	1.07 (1.01, 1.13)	1.06 (1.01, 1.12)
Magnitude of first blip (reference no blips)		
Low (50 - 199 copies/mL)	1.15 (0.79, 1.66)	1.00 (0.72, 1.40)
Medium (200 - 499 copies/mL)	1.34 (0.71, 2.51)	1.22 (0.73, 2.02)
High (500 - 999 copies/mL)	1.42 (0.87, 2.33)	1.24 (0.78, 2.00)

[a] Adjusted for the same covariates as in Table 2: gender, transmission by injection drug use, age at the start of the suppression episode, the year the suppression episode began, the assay used to measure the blip, and time updated cART categories. The gap-time model also included covariates for the yearly rate of viral load measurements (as in [1]); in the model for interval censored data, these three covariates were dropped and two covariates were added to represent time updated CD4 cell count.

References

1. Grennan JT, Loutfy MR, Su D, Harrigan PR, Cooper C, Klein M, et al. Magnitude of virologic blips is associated with a higher risk for virologic rebound in HIV-infected individuals: a recurrent events analysis. J Infect Dis 2012; 205:1230-8.

2. Panel on Antiretroviral Guidelines for Adults and Adolescents. Guidelines for the use of antiretroviral agents in HIV-1-infected adults and adolescents. http://aidsinfo.nih.gov/contentfiles/lvguidelines/adultandadolescentgl.pdf (2014). Accessed 10 June 2014.

3. Smith PJ, Thompson TJ, Jereb JA. A model for interval-censored tuberculosis outbreak data. Stat Med 1997; 16:485-96.
